# Supplementary material for: Distinctive properties of the prion protein in the brain and retina in the amyloidosis associated with the PRNP F198S mutation
Source: Acta Neuropathol. 2026 Aug 26;152(1):25. doi: 10.1007/s00401-026-03060-z (PMC13518383; doi:10.1007/s00401-026-03060-z)
Supplement: Supplementary file 1 [file 401_2026_3060_MOESM1_ESM.pdf]

Supplemental File

Distinctive properties of the prion protein in the brain and retina in the amyloidosis associated with the *PRNP* F198S Mutation

Acta Neuropathologica

Bernardino Ghetti[1], Bradley S. Glazier[1], Michele Fiorini[2], Max Jacobsen[1],Kathy L. Newell[1], José M. Bonnin[1], Jill R. Murrell[1], Leah Rie Varner[3], James F. Striebel[3], Suzette A. Priola[3], Gianluigi Zanusso[2]

Corresponding author:
Bernardino Ghetti, MD
Department of Pathology & Laboratory Medicine
Indiana University School of Medicine
635 Barnhill Drive, MS A138
Indianapolis, IN 46202, USA
Phone: (317) 339-4700
Email: bghetti@iu.edu

Supplemental Genetic and Clinical Data

Genetics

The eight individuals, who had been clinically diagnosed as being affected by GSS, were all members of a large kindred, that has been previously described. Sequencing of DNA extracted *postmortem* from brain tissue of all eight subjects for DNA analysis of *PRNP*, revealed a single nucleotide thymine to cytosine substitution at codon 198 (c.593T>C) in one allele, resulting in a phenylalanine to serine amino acid change (p. Phe198Ser). Subjects A, F, and G were heterozygous methionine/valine at residue 129 (c.385A>G, p. Met129Val), while subjects B, C, D, E, and H were homozygous valine/valine.

Clinical Highlights

The clinical phenotype in all patients was characterized by progressive impairment of cognition, behavioral symptoms, cerebellar signs, such as clumsiness, ataxia and dysarthria, parkinsonism and pyramidal signs. Clinical data on subjects A, E, F, G and H have been previously reported. No major visual deficit was described. A clinical history from each patient is reported below.

Anamnestic Data of Individuals included in this study

Subject A

Age at Initial Neurological Exam:	51 years
Disease Duration:	7 years
Age at Death:	58 years
Birth Sex:	Male
Neuropathologic Diagnosis:	GSS
Molecular Genetic Mutation:	F198S
PrP 129 Polymorphism:	M/V

Clinical Signs & Symptoms at Onset of Clinical Signs:

Age 51: Asymmetry heel to shin test, dystaxic finger-to-nose, swallowing difficulty

Clinical Signs & Symptoms During Disease Progression:

Age 52: Neuropsychology: decline in psychomotor speed; Neurologic data (informant and exam): irritability, slurred speech, depression

Age 53: Neuropsychology: decline in new learning efficiency, spatial processing, manual dexterity. Conclusion: Mild subcortical dementia. Neuropsychology: decline in fluency, new learning, mental flexibility, manual dexterity. Neurological exam: Rigidity, bradykinesia, impaired speech and facial expression, masked facies, nystagmus in lateral gaze. Behavioral scale: agitation, depression, apathy, disinhibition, irritability, nighttime behavior.

Age 54: Neuropsychology: decline in fluency, new learning efficiency, spatial processing, manual dexterity. Impaired functions of daily living. Neurological exam: Progression of signs and symptoms. Rigidity, bradykinesia, impaired speech and facial expression, masked facies, shuffling gait, tremor. Behavioral scale: agitation, depression.

Age 55: Memory and cognitive decline over past few years. Clumsiness and slowing of movements. Difficulty with swallowing. Agitation, Neurological exam: Masked facies. Lateral gaze nystagmus, dysdiadochokinesia, rigidity, bradykinesia, cogwheel lower and upper extremities, asymmetric heel to shin test, no tremor, ataxia of gait.

Age 56: Progression of all symptoms. Presence of tremor, jerks, shuffling gait.

Age 57: Further progression. Dementia, predominantly of executive type, with parkinsonian and cerebellar signs.

Age 58: Nursing home. Wheelchair-bound. Skin breakdown. Respiratory

Subject B

Age at Initial Neurological Exam:	45 years
Disease Duration:	3 years
Age at Death:	48 years
Birth Sex:	Male
Neuropathologic Diagnosis:	GSS
Molecular Genetic Mutation:	F198S
PrP 129 Polymorphism:	V/V

Clinical Signs & Symptoms at Onset of Clinical Signs:

Age 45: Personality change, aggressive and inappropriate behavior, abusive

Clinical Signs & Symptoms During Disease Progression:

Age 46, Age 47: Anxiety, withdrawn. Motor domain: slow movement; unstable shuffling gait. Decreased facial expression. Language domain: word finding difficulty. Cognitive domain: forgetful, unable to manage finances. MMSE 24/30. Multidomain Impairment. Neurological exam: Rigidity, bradykinesia, no tremor, dysdiadochokinesia, asymmetric heel to shin test, stooped posture.

Subject C

Age at Initial Neurological Exam:	31 years
Disease Duration:	13 years
Age at Death:	44 years
Birth Sex:	Female
Neuropathologic Diagnosis:	GSS
Molecular Genetic Mutation:	F198S
PrP 129 Polymorphism:	V/V

Clinical Signs & Symptoms at Onset of Clinical Signs:

Age 31: Intermittent numbness of hands and arms; tingling of hands, right foot, head. Intermittent dysarthria. Neurological exam: Finger-to-nose mildly dystaxic on left side; left upper extremity dysdiadochokinesia. Cognitive domain: Mild cognitive dysfunction, impaired attention, anterograde memory, psychomotor slowing.

Clinical Signs & Symptoms During Disease Progression:

Age 33: Neurological exam: Finger-to-nose mildly dystaxic on left side. Abnormal heel to shin test.

Age 39: Memory has worsened; unable to manage finances. Clumsiness of arms and legs. Motor domain. Asymmetric heel to shin test, broad based walk. Cognitive domain: forgetful, needs supervision. Mild dementia.

Age 40: Decline in areas of fluency, visual memory, spatial reasoning, complex sequential tracking. Moderate dystaxia upper and lower extremities.

Age 42: Progression of ataxia and cognitive decline. Generalized weakness. Tremor, shaking of upper extremities. Carbidopa/ levodopa improve symptoms.

Age 43: Memory has worsened. Language issues.

MRI:

Age 40: General cerebral atrophy, atrophy caudate nucleus, cerebellum

Other:

Age 40: FDG PET: Frontal hypometabolism;

Age 40: Amyloid PET: negative

Subject D

Age at Initial Neurological Exam:	45 years
Disease Duration:	4 years
Age at Death:	49 years
Birth Sex:	Female
Neuropathologic Diagnosis:	GSS
Molecular Genetic Mutation:	F198S
PrP 129 Polymorphism:	V/V

Clinical Signs & Symptoms at Onset of Clinical Signs:

Age 48: History of depression, anxiety, frequent falls. Neurological exam: rigidity, tremor upper extremities, cogwheel lower and upper extremities, hyperreflexia, ataxia of gait, dysarthria, dysphagia. Severe cognitive decline. Psychiatric evaluation: depression, suicidal ideation, paranoid thinking.

Clinical Signs & Symptoms During Disease Progression:

Subject E

Age at Initial Neurological Exam:	48 years
Disease Duration:	5 years
Age at Death:	53 years
Birth Sex:	Male
Neuropathologic Diagnosis:	GSS
Molecular Genetic Mutation:	F198S
PrP 129 Polymorphism:	V/V

Clinical Signs & Symptoms at Onset of Clinical Signs:

Age 48: At least one-year history of gradually progressive memory and language disorder and a six-month history of difficulties with judgment and reasoning, paranoid ideation, changes in personality: irritability, withdrawal, sadness, socially inappropriate behavior, and agitation. Neuropsychology tests: mild impairment in memory, orientation, judgement, problem-solving, community affairs, home and hobbies domains, moderate impairment in the behavior, comportment, and personality domains. presence of agitation, depression, anxiety, disinhibition, irritability, nighttime behaviors, problems with appetite, impairment in daily functions. The neuropsychological battery revealed mild impairments in efficiency of new learning, verbal fluency, response inhibition, complex sequential tracking, and manual motor speed. Neurological exam: rigidity, bradykinesia, body and extremities

Clinical Signs & Symptoms During Disease Progression:

Age 52: Neurological exam: rigidity, bradykinesia, body and extremities, ataxia of gait, dysdiadochokinesia, asymmetric heel to shin test, stooped posture. Neuropsychology tests: mild impairment in memory, orientation, judgement, problem-solving. Severe progression of symptoms.

MRI:

Age 52: Global atrophy

Other:

Age 52: PET flortaucipir: show specific binding of the tracer in the basal ganglia, thalamus, cingulate gyrus, frontal lobe, and insula/claustrum [2]

Subject F

Age at Initial Neurological Exam:	68 years
Disease Duration:	5 years
Age at Death:	73 years
Birth Sex:	Female
Neuropathologic Diagnosis:	GSS
Molecular Genetic Mutation:	F198S
PrP 129 Polymorphism:	M/V

Clinical Signs & Symptoms at Onset of Clinical Signs:

Age 68: Unsteady gait, rigidity, cognitively impaired.

Clinical Signs & Symptoms During Disease Progression:

Age 72: Neurological exam: Rigidity, bradykinesia, dysarthric and facial expression, masked facies, difficulty upward gaze. Cognitively impaired.

Age 73: Neurological exam: Rigidity, bradykinesia, impaired speech and facial expression, masked facies, nystagmus. Contractures of arms and legs. Cognitively impaired.

MRI:

Age 72: Diffuse cortical atrophy, ischemic changes

Other:

Age 72: Eye Exam: Eye movement abnormalities consistent with cerebellar pathology [3]

Subject G

Age at Initial Neurological Exam:	50 years
Disease Duration:	13 years
Age at Death:	63 years
Birth Sex:	Female
Neuropathologic Diagnosis:	GSS
Molecular Genetic Mutation:	F198S
PrP 129 Polymorphism:	M/V

Clinical Signs & Symptoms at Onset of Clinical Signs:

Age 32: Sleep disorder

Age 35: Depression, suicidal thoughts

Age 54: Left sided balance problem, memory problems

Clinical Signs & Symptoms During Disease Progression:

Age 57: Memory, problem with executive function, word finding difficulties, unstable gait with multiple falls, tremor

Age 58: Brisk reflexes, unstable gait, Neuropsychological Exam: Subcortical MCI, affecting executive cognitive function, motor skills, and mood.

Age 59: Intention tremor with dysmetria, bilateral digital myoclonus, ataxic gait, mild degree of disinhibition. Neuropsychological Exam: mild impairment in memory and learning, verbal fluency, manual motor function, executive cognitive function, mild depression.

Age 61: Neuropsychological Exam: mild progression

Age 62: Neuropsychological Exam: mild progression

MRI:

Age 53: Mild cerebral volume loss
Age 58: Mild cerebral volume loss progressing since prior MRI.

Other:

Age 57: PET flortaucipir: specific binding of the tracer in the basal ganglia, thalamus, insular cortex, postcentral gyrus, and inferior temporal lobe [2].

Subject H

Age at Initial Neurological Exam:	47 years
Disease Duration:	13 years
Age at Death:	60 years
Birth Sex:	Female
Neuropathologic Diagnosis:	GSS
Molecular Genetic Mutation:	F198S
PrP 129 Polymorphism:	V/V

Clinical Signs & Symptoms at Onset of Clinical Signs [1]

Age 47-51: Progressive increasing unsteadiness of gait, falls, speech less intelligible, difficulty remembering names.

Age 51: Neurologic examination: unable to subtract serial 7's from 100, impaired pursuit eye movements, bilateral difficulty with heel-to-shin testing, upper extremity rapid alternating movements, broad-based and ataxic gait, verbal IQ of 88, ideokinetic apraxia, bilateral finger agnosia.

Age 54: Worsened gait dystaxia, incoordination of upper extremities, very dysarthric speech, mental confusion,

Age 55-60: Cogwheel rigidity and progressive worsening of ataxia and cognitive symptoms. Bedbound

MRI:

Age 57: Cerebellar atrophy

Other:

Age 57: Eye exam: abnormal eye movements characteristic of extrapyramidal diseases and cerebellar disorders [3].

Subject I

Age at Initial Neurological Exam:	42 y
Disease Duration:	5 y
Age at Death:	45 y
Birth Sex:	Female
Neuropathologic Diagnosis:	GSS
Molecular Genetic Mutation:	A117V
PrP 129 Polymorphism:	M/V

Clinical Signs & Symptoms at Onset of Clinical Signs [1]

Age 40 (family reported): Handwriting issues and memory problems.

Age 41 (family reported): Word finding difficulty

Age 42, Visit 1: Difficulty with word finding, quicker to anger, MVA but no mention of if loss of conscious. Neurologist noted mild tremor in the right hand, but otherwise normal exam. Pt did cry frequently during the exam. MRI was dx'd as "negative MRI" (I presume means normal).

Age 42, Visit 2: Mild right hand tremor. Diminished reflexes throughout. Lower extremity weakness. Left leg limping in her gait. CDR of 1, sum of boxes was 11. No Parkinson signs.

Control 1

Age at Initial Neurological Exam: 57 years
Disease Duration: 10 years
Age at Death: 67 years
Birth Sex: Male
Neuropathologic Diagnosis: Diffuse Lewy Body Disease

Clinical Signs & Symptoms at Onset of Clinical Signs:
Age 51-57: Attentional and visuospatial deficits, the emergence of visual hallucinations, rigidity, cognitive decline, withdrawn, slowness of movement, shuffling gait, intermittent upper extremity rest tremor, micrographia, stooped posture, hypophonia, sleep apnea.

Clinical Signs & Symptoms Progression
Age 57: Diagnosed with dementia with Lewy bodies in the mild stage

Age 59: Well-formed visual hallucinations, cognitive decline, parkinsonism, postural tremor in the upper extremities bilaterally, retropulsion

Age 60: Advancing memory function, persistence of visual hallucinations, significant parkinsonism, treated Sinemet.

Control 2

Age at Initial Neurological Exam: 57 Years
Disease Duration: 9 Years
Age at Death: 66 Years
Birth Sex: Female
Neuropathologic Diagnosis: Alzheimer disease

Clinical Signs & Symptoms at Onset of Clinical Signs:

Age 57: Episodic memory disturbance. Clinical diagnosis: MCI amnestic

Clinical Signs & Symptoms Progression:

Age 62: Multiple cognitive Symptoms at evolution: Attentional, Dis-executive and visuospatial deficits. Clinical diagnosis: Alzheimer disease

Age 63: Epileptic Seizures. The patient was bedridden in akinetc mutism

Age 65: Pneumonia

MRI:

Age 57: Brain atrophy;

SPECT:

Age 57: hypometabolism in medial temporal lobe bilaterally.

Control 3

Age at Initial Neurological Exam: 67 Years
Disease Duration: 3 Months
Age at Death: 67 Years
Birth Sex: Male
Neuropathologic Diagnosis: Creutzfeldt-Jakob Disease

Clinical Signs & Symptoms at Onset of Clinical Signs:

Age 67: Cognitive disturbances (Visuospatial and hallucinations) and ataxia. CSF analysis: RT-QuIC negative. Olfactory mucosa: RT-QuIC positive.

Clinical Signs & Symptoms Progression

Age 67: Akinetic mutism and myoclonus

MRI:

Age 67: hyperintense signals in basal ganglia and cortical ribbon (temporo-parietal-occipital).

EEG:

Age 67: period triphasic, sharp, and slow waves.

REFERENCES

1. Farlow, M.R., R.D. Yee, S.R. Dlouhy, P.M. Conneally, B. Azzarelli, and B. Ghetti, *Gerstmann-Straussler-Scheinker disease. I. Extending the clinical spectrum*. Neurology, 1989. **39**(11): p. 1446–52.DOI: 10.1212/wnl.39.11.1446.
2. Risacher, S.L., M.R. Farlow, D.R. Bateman, F. Epperson, E.F. Tallman, R. Richardson, J.R. Murrell, F.W. Unverzagt, L.G. Apostolova, J.M. Bonnin, B. Ghetti, and A.J. Saykin, *Detection of tau in Gerstmann-Straussler-Scheinker disease (PRNP F198S) by [(18)F]Flortaucipir PET*. Acta Neuropathol Commun, 2018. **6**(1): p. 114.DOI: 10.1186/s40478-018-0608-z.
3. Yee, R.D., M.R. Farlow, D.A. Suzuki, K.F. Betelak, and B. Ghetti, *Abnormal eye movements in Gerstmann-Straussler-Scheinker disease*. Arch Ophthalmol, 1992. **110**(1): p. 68–74.DOI: 10.1001/archopht.1992.01080130070028.
